# Interpreting Social Accounting Matrix (SAM) as an Information Channel

**DOI:** 10.3390/e22121346

**Published:** 2020-11-28

**Authors:** Mateu Sbert, Shuning Chen, Miquel Feixas, Marius Vila, Amos Golan

**Affiliations:** 1 Graphics and Imaging Laboratory, University of Girona, 17003 Girona, Spain; feixas@ima.udg.edu (M.F.); marius.vila@gmail.com (M.V.); 2 Research Institute of Innovative Technology for the Earth, Kyoto 6190292, Japan; chensn@rite.or.jp; 3 Department of Economics, American University, Washington, DC 20016, USA; agolan@american.edu; 4 Sante Fe Institute, Albuquerque, NM 87501, USA

**Keywords:** social accounting matrix, entropy, mutual information, information channel, markov chain

## Abstract

Information theory, and the concept of information channel, allows us to calculate the mutual information between the source (input) and the receiver (output), both represented by probability distributions over their possible states. In this paper, we use the theory behind the information channel to provide an enhanced interpretation to a Social Accounting Matrix (SAM), a square matrix whose columns and rows present the expenditure and receipt accounts of economic actors. Under our interpretation, the SAM’s coefficients, which, conceptually, can be viewed as a Markov chain, can be interpreted as an information channel, allowing us to optimize the desired level of aggregation within the SAM. In addition, the developed information measures can describe accurately the evolution of a SAM over time. Interpreting the SAM matrix as an ergodic chain could show the effect of a shock on the economy after several periods or economic cycles. Under our new framework, finding the power limit of the matrix allows one to check (and confirm) whether the matrix is well-constructed (irreducible and aperiodic), and obtain new optimization functions to balance the SAM matrix. In addition to the theory, we also provide two empirical examples that support our channel concept and help to understand the associated measures.

## 1. Introduction

In 1948, Claude E. Shannon (1916–2001) published “A mathematical theory of communication” [1] which established the basic concepts of information theory, such as entropy and mutual information. These notions have been widely used in many fields, such as physics, computer science, neurology, image processing, computer graphics, and visualization. Shannon also introduced the concept of *communication* or *information channel*, to model the communication between source and receiver. This concept is general enough to be applied to any two variables sharing information. In an information channel, the source (or input) and receiver (or output) variables are defined by a probability distribution over their possible states and are related by an array of conditional probabilities. These probabilities define the different ways that a state in the output variable can be reached from the states in the input variable. In short, the channel specifies how the two variables share, or transfer, information. The input and output variables can be of any nature, they can be defined or not on the same states and they can be even the same.

Here, the concept of information channel will be applied to the Social Accounting Matrix (SAM), a square matrix whose corresponding columns and rows present the expenditure and receipt accounts of economic actors [2]. Social accounting matrixes (SAM) are used often to study the economy of a country or a region. They capture the complete information about all (at the relevant level of resolution) transactions between economic agents in a specific economy for a specific period of time. Broadly speaking, they extend the classical Input-Output framework, including the complete circular flow of income in the economy [3]. SAM matrixes have been recently used to study regional economic impact of tourism [4], carbon emission [5], the role of bioeconomy [6], the environmental impacts of policies [7], and key sectors in regional economy [8]. The significance of our contribution is in its new powerful tools that extend the understanding of SAM’s. To the best of our knowledge our development and interpretation is new.

In this paper, we provide a new tool for analyzing an economic system. We show that the SAM coefficients matrix can be thought of as an ergodic Markov chain, and subsequently can be represented as an information (or communication) channel. Both ergodic Markov chain and information channels are well studied in information theory. SAM’s are studied in economics and at times are used in other disciplines. Our study combines the tools of information theory and the tools of balancing, designing and understanding SAM’s. Our interpretation of the matrix of SAM’s coefficients fits into the state of the art balancing techniques, and opens a whole new insight into the meaning and understanding of SAM’s. Under our interpretation, the SAM’s coefficients, which are associated with a Markov chain and the information channel, can be interpreted as information-theoretic quantities. That allows us to optimize the desired level of aggregation, to quantify the ’closeness’ of sectors within the SAM, as well as provide new interpretations to the coefficients and the matrix as a whole. The set of information measures can describe quite precisely the evolution of a SAM time series. Interpreting the SAM matrix as an ergodic chain could show the effect of a shock on the economy after several periods or economic cycles. Under our new framework, finding the power limit of the matrix allows one to check (and confirm) that the matrix is well-constructed. Based on the information channel model, new optimization functions to fill missing SAM coefficients can be obtained.

The rest of this paper is organized as follows. In Section 2, we present the basics on information measures and information channel, and interpret a Markov chain as an information channel. In Section 3, we present the SAM matrix as an ergodic Markov chain first and then as an information channel. In Section 4 we show how the cross entropy method used to fill the unknowns in the SAM matrix fits well into the information channel model. In Section 5 we show several examples of our model, and in Section 6 we present our conclusions and future work. Finally, we add a toy example in Appendix A that follows step by step how to obtain from a toy 3 × 3 SAM matrix a Markov chain and an information channel with all associated mesures.

## 2. Information Measures and Information Channel

In this section, we briefly describe the most basic information-theoretic measures [9,10,11], the main elements of an information channel [9,10], and a Markov chain as an information channel.

### 2.1. Basic Information-Theoretic Measures

Let *X* be a discrete random variable with alphabet X and probability distribution {p(x)}, where p(x)=Pr{X=x} and x∈X. The distribution {p(x)} can also be denoted by p(X). Likewise, let *Y* be a random variable taking values *y* in Y.

Following Hartley [12], Shannon assigned to each possible result *x* an uncertainty (before the realization of *X*) or an information content (after the realization of *X*) of $\log\frac{1}{p(x)}$. Then *Shannon entropy*H(X) was defined by

(1) $$H\left(X\right)=-\sum_{x\in X}p\left(x\right)\log p\left(x\right),$$

where logarithms are taken in base 2 and then entropy is measured in bits. We use the convention 0log0=0. H(X), denoted as H(p) too, measures the average *uncertainty* or information content of a random variable *X*. The maximum value of H(X), $\log_{2}n$, happens for the uniform distribution, when for all *x* all probabilities are equal, $p\left(x\right)=\frac{1}{n}$ where n=card(X). The minimum value of H(x) is 0, when for some *x*, p(x) = 1 and all other probabilities are thus 0. Thus, entropy can be considered too a measure of homogeneity or uniformity of a distribution [13] or a diversity index [14], the higher its value the more homogeneous is the distribution and vice versa.

One important property of entropy is the *grouping property*. Suppose we merge two indexes, which without loss of generality we can consider first and second index, then

(2) $$H\left(p\right)=H\left(p^{\prime }\right)+\left(p_{1}+p_{2}\right)H\left(\frac{p_{1}}{p_{1}+p_{2}},\frac{p_{2}}{p_{1}+p_{2}}\right),$$

where $p^{\prime }=\left\{p_{1}+p_{2},p_{3},...\right\}$. This property can be generalized to grouping any number of indexes. From Equation (2) we see that entropy holds the *coarse grain property*, which states that index grouping implies a loss of entropy:

(3) $$H\left(p\right)\geq H\left(p^{\prime }\right).$$

Coarse grain property tells us that when we lose detail we lose information.

The *conditional entropy* H(Y|X) is defined by

(4) $$H\left(Y|X\right)=\sum_{x\in X}p\left(x\right)H\left(Y|x\right),$$

where p(y|x)=Pr[Y=y|X=x] is the conditional probability and $H\left(Y|x\right)=-\sum_{y\in Y}p\left(y|x\right)\log p\left(y|x\right)$ is the entropy of *Y* given *x*.

H(Y|X) measures the average uncertainty associated with *Y* if we know the outcome of *X*.

The *relative entropy* or *Kullback-Leibler (or K-L) distance or divergence* $D_{KL}\left(p,q\right)$ between probability distributions *p* and *q*, defined over the same alphabet X, is given by

(5) $$\begin{array}{c} D_{KL}\left(p,q\right)=\sum_{x\in X}p\left(x\right)\log\frac{p(x)}{q(x)}. \end{array}$$

We adopt the conventions that 0log(0/0)=0 and alog(a/0)=∞ if a>0. The Kullback-Leibler distance holds the coarse grain property Equation (3) too (which for divergences is a particular case of the *data processing inequality* [9,15]): if we group indexes in the alphabet X so that we obtain a new simplified alphabet $X^{\prime }$ and probability distributions $p^{\prime }$ and $q^{\prime }$, which are obtained from *p* and *q* by adding the probability values of the grouped indexes, then

(6) $$\begin{array}{c} D_{KL}\left(p,q\right)=\sum_{x\in X}p\left(x\right)\log\frac{p(x)}{q(x)}\geq D_{KL}\left(p^{\prime },q^{\prime }\right)=\sum_{x\in X}p^{\prime }\left(x\right)\log\frac{p^{\prime }\left(x\right)}{q^{\prime }\left(x\right)}. \end{array}$$

The reverse is also true, i.e., if we refine the indexes we increase the K-L distance between the distributions.

The *mutual information* I(X;Y) between *X* and *Y* is defined by

(7) $$\begin{array}{ccc} I(X;Y) & = & H(Y)-H(Y|X) \end{array}$$

(8) $$\begin{array}{ccc} & = & \sum_{x\in X}\sum_{y\in Y}p\left(x,y\right)\log\frac{p(x,y)}{p(x)p(y)}, \end{array}$$

(9) $$\begin{array}{ccc} & = & D_{KL}\left(p\left(X,Y\right),p\left(X\right)p\left(Y\right)\right) \end{array}$$

where p(x,y)=Pr[X=x,Y=y] is the joint probability. From Equation (8), mutual information is symmetrical, i.e., I(X;Y)=I(Y;X). Mutual information expresses the *shared information* between *X* and *Y*. Observe that being a K-L distance, it holds the data processing inequality, whenever we cluster (or refine) on X or Y (or both simultaneously) indexes. This is, if $X^{\prime },Y^{\prime }$ are the resulting random variables on the clustered domains $X^{\prime },Y^{\prime }$ then

(10) $$\begin{array}{c} I\left(X;Y\right)\geq I\left(X^{\prime };Y^{\prime }\right) \end{array}$$

This fact is used in the *information bottleneck method*, introduced by Tishby et al. [16], which aims at clustering with minimum loss of mutual information or at refining with maximum gain of mutual information.

**Figure 1:**
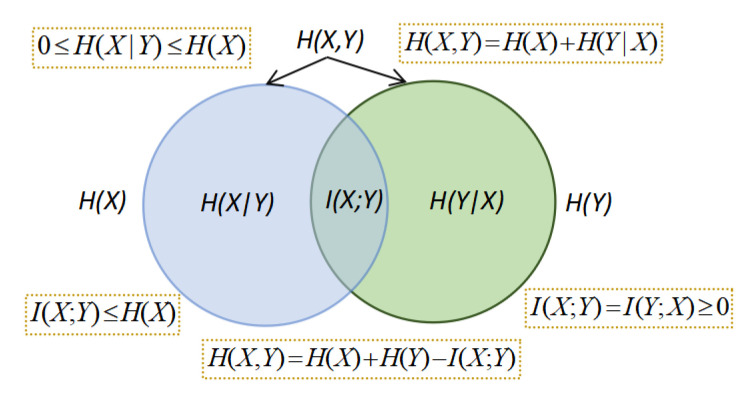
The information diagram shows the relationship between Shannon’s information measures. Using an analogy to a Venn’s set diagram, the intersection of sets in blue (entropy of *X*, H(X)), and green, (entropy of *Y*, H(Y), is the mutual information I(X;Y). Their union is the joint entropy H(X,Y). The set H(X) minus the set H(Y) is the conditional entropy H(X|Y). The equalities and inequalities shown in the diagram can be directly deduced from Venn’s set analogy.

The relations between Shannon’s information measures are summarized in the information diagram of Figure 1 [10].

We also present another information quantity that will be discussed further in Section 3.2 and will be useful in Section 4. The *cross entropy* CE(X,Y) of random variables X,Y with distributions p,q respectively is defined as

(11) $$\begin{array}{c} CE\left(X,Y\right)=-\sum_{i}p_{i}\log q_{i} \end{array}$$

It can be easily seen that

(12) $$\begin{array}{c} CE\left(X,Y\right)=H\left(X\right)+D_{KL}\left(X,Y\right) \end{array}$$

As entropy and Kullback-Leibler distance are always positive, cross entropy is always positive too. The minimum cross entropy happens when X≡Y, where $D_{KL}\left(X,Y\right)=0$ and thus CE(X,Y)=H(X).

### 2.2. Information Channel

Conditional entropy H(Y|X) and mutual information I(X;Y) can be thought of in the context of a *communication channel* or *information channel*X→Y whose output *Y* depends probabilistically on its input *X* [9]. They express the uncertainty in the channel output from the sender’s point of view, H(Y|X), and the degree of dependence or information transfer in the channel between variables *X* and *Y*, I(X;Y).

**Figure 2:**
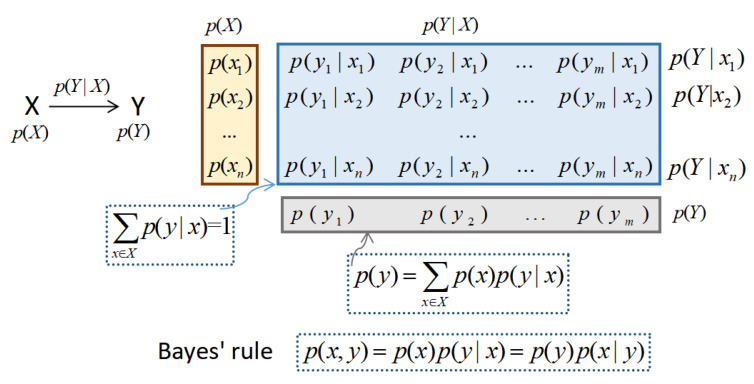
Main elements of an information channel X→Y. Input and output variables, *X* and *Y*, with their probability distributions p(X) and p(Y), and probability transition matrix p(Y|X), composed of conditional probabilities p(y|x). They are related by the equation p(Y)=p(X)p(Y|X), which determines the output distribution p(Y) given the input distribution p(X). All these elements are connected by Bayes’ rule.

The diagram in Figure 2 shows the elements of an information channel. These elements are:

- Input and output variables, *X* and *Y*, with probability distributions p(X) and p(Y), called marginal probabilities.
- Probability transition matrix p(Y|X) (with elements conditional probabilities p(y|x)) determining the output distribution p(Y) given the input distribution p(X): $p\left(y\right)=\sum_{x\in X}p\left(x\right)p\left(y|x\right)$. Each row of p(Y|X), denoted by p(Y|x), is a probability distribution.

All these elements are connected by Bayes’ rule that relates marginal (input and output), conditional, and joint probabilities: p(x,y)=p(x)p(y|x)=p(y)p(x|y).

Well-known applications of information channels are found in the fields of visual computing [17], image registration channel and stimulus-response channel. Registration between two images can be modeled by an information channel, where its marginal and joint probability distributions are obtained by simple normalization of the corresponding intensity histograms of the overlap area of both images [18,19], under the conjecture that the optimal registration corresponds to the maximum mutual information between the overlap areas of the two images. In the stimulus-response channel, mutual information between stimulus and response quantifies how much information the neural responses carry about the stimuli, i.e., the information shared or transferred between stimuli and responses [20,21], and also the specific information associated with each stimulus (or response).

### 2.3. A Markov Chain as an Information Channel

A Markov discrete random walk [22] is characterized by the transition probabilities between the states. These probabilities form a so-called stochastic matrix, *P*, where for all i,j, $p_{ij}\geq 0$, and $\sum_{j}p_{ij}=1$. If $\lim_{n->\infty }P^{n}$ exists, the *equilibrium distribution*π exists and it holds

(13) π=πP.

The $\lim_{n->\infty }P^{n}$ is formed by rows all equal to π. For all *i*, $\pi_{i}$ gives the fraction of the total of visits a random walk has visited state *i*.

Any distribution holding Equation (13) is called a *stationary distribution*. If the equilibrium distribution exists it is the unique stationary distribution. On the other hand, the fact that there exists a stationary distribution does not mean that it is the equilibrium distribution. To put a simple example, consider P={{0,1},{1,0}}. The distribution π={1/2,1/2} is stationary, but there is no equilibrium distribution as $P^{n}$ oscillates and thus $\lim_{n->\infty }P^{n}$ does not exist.

The equilibrium distribution exists when the Markov chain is *irreducible and aperiodic*. Irreducible means that every state can be reached from every one else after a finite number of applications of the transition matrix *P*. This is, all states communicate with each other after several transitions. If not, when for instance there were an absorbing state, or a set of states which can be reached but cannot be exited, the Markov chain would be reducible, and the states can be divided into equivalence class, where all states in one class communicate with each other. An irreducible Markov chain contains thus a single class of equivalence.

A state is periodic when we can only return to it by several transitions multiple of some integer >1 which is called the period of the state. When there is no periodic state the Markov chain is aperiodic. All states of an irreducible Markov chain have the same period [23]. An irreducible and aperiodic Markov chain is also called *ergodic*.

Any Markov chain with transition probabilities matrix *P* and stationary distribution π, this is, holding Equation (13), can be interpreted as an information channel, with X=Y, and p(X)=p(Y)=π, and p(Y|X)=P. Observe that we do not need ergodic property for a Markov chain to be interpreted as an information channel, although indeed it is a desirable property. We will justify in the next Section that the SAM coefficients matrix is an ergodic Markov chain, which will be corroborated with the examples considered.

#### 2.3.1. Grouping Indexes

Suppose we want to group indexes, simultaneously in input and output, so that (X,Y) becomes $\left(X^{\prime },Y^{\prime }\right)$. How does the matrix P=p(Y|X) transform so that we have a channel with new transition matrix $P^{\prime }=p\left(X^{\prime }|Y^{\prime }\right)$, $p\left(X^{\prime }\right)=p\left(Y^{\prime }\right)=\pi^{\prime }$? We just have to use the joint probabilities p(x,y) that if they are not known a priori can be obtained from conditional and marginal probabilities by Bayes theorem p(x,y)=p(x)p(y|x)=p(y)p(x|y). We obtain the new joint probabilities $p(x^{\prime },y^{\prime })$ by adding over the grouped indexes, first by row and then by column or vice versa, and the new marginals are $p\left(x^{\prime }\right)=\sum_{y^{\prime }\in Y^{\prime }}p\left(x^{\prime },y^{\prime }\right),p\left(y^{\prime }\right)=\sum_{x^{\prime }\in X^{\prime }}p\left(x^{\prime },y^{\prime }\right)$, and the new conditional probabilities are $p\left(y^{\prime }|x^{\prime }\right)=p\left(x^{\prime },y^{\prime }\right)/p\left(x^{\prime }\right)$, $p\left(x^{\prime }|y^{\prime }\right)=p\left(x^{\prime },y^{\prime }\right)/p\left(y^{\prime }\right)$. By construction, and because p(X)=p(Y), the new marginals hold $p\left(X^{\prime }\right)=p\left(Y^{\prime }\right)=\pi^{\prime }$, $\pi^{\prime }=\pi^{\prime }P^{\prime }$, with $P^{\prime }=p\left(X^{\prime }|Y^{\prime }\right)$. Observe that grouping keeps the ergodicity, this is, if *P* is ergodic, $P^{\prime }$ will be ergodic too.

All in all, when we group rows (and respective columns) and pass from X,Y to $X^{\prime },Y^{\prime }$, we have that the following grouping inequalities (see Section 3.2.1 too for grouping of mutual information) hold in our channel:

(14) $$\begin{array}{c} H\left(X\right)\geq H\left(X^{\prime }\right) \end{array}$$

(15) $$\begin{array}{c} H\left(X,Y\right)\geq H\left(X^{\prime },Y^{\prime }\right) \end{array}$$

(16) $$\begin{array}{c} I\left(X;Y\right)\geq I\left(X^{\prime };Y^{\prime }\right) \end{array}$$

#### 2.3.2. Dual Channel

In this section, we considered the channel X→Y, with p(X)=p(Y)=π and conditional probabilities p(Y|X)=P, and thus p(Y)=p(X)p(Y|X), or π=πP. However, using Bayes’s theorem we can compute the conditional probabilities $p\left(X|Y\right)=P^{d}$ for inverse or *dual* channel Y→X, and then p(X)=p(Y)p(X|Y), or $\pi =\pi P^{d}$. Observe that $P^{d}$ is also a ergodic Markov chain with the same equilibrium distribution π. Indeed we have H(X)=H(Y), and also H(X|Y)=H(Y|X), and joint entropy and mutual information are equal. The differences between the two channels will be found in the entropy and mutual information of rows. Observe that given the joint distribution matrix $\left\{p\left(x_{i},y_{j}\right)\right\}$ and the marginals $\left\{p\left(x_{i}\right)=p\left(y_{i}\right)=\pi_{i}\right\}$, we obtain the respective conditional probabilities $\left\{p\left(y_{j}|x_{i}\right)=\frac{p(x_{i},y_{j})}{p(x_{i})}=\frac{p(x_{i},y_{j})}{\pi_{i}}\right\}$ and $\left\{p\left(x_{i}|y_{j}\right)=\frac{p(x_{i},y_{j})}{p(y_{j})}=\frac{p(x_{i},y_{j})}{\pi_{j}}\right\}$, i.e., the normalized rows of the joint distribution will form the p(Y|X)=P matrix, and the normalized columns (once transposed) the $p\left(X|Y\right)=P^{d}$ matrix. Indeed, ${\left(P^{d}\right)}^{d}=P$.

## 3. SAM Matrix

In this section, we show how a SAM matrix can be built to an Information channel, by considering it first an ergodic Markov chain and then interpreting this chain as an information channel.

### 3.1. SAM Coefficient Matrix as a Markov Chain

The Social Accounting Matrix (SAM), represents all monetary flows in an economy, from sources to recipients. Given a SAM matrix *T*, the element $t_{ij}$ represents the amount of money from state *j* to state *i* (we will use in this paper synonymously the words state, that comes from Markov chain literature, economic actor, account, sector). The vector of totals, *y*, is such that $y_{j}=\sum_{i}t_{ij}=\sum_{i}t_{ji}$, this is, rows and columns sum equal. This is because the total amount of money received by sector *i* has to be equal to the total amount spent. The SAM coefficient matrix *A* is defined as $a_{ij}=t_{ij}/y_{j}$. By construction, the SAM coefficient matrix and vector of totals *y* hold y=Ay, and considering normalized y vector, we have too $y_{i}=y_{i}/\sum_{j}y_{j}$, y=Ay. Observe that *A* cannot be considered a stochastic, or conditional probability, matrix, this is, in general $\sum_{j}a_{ij}=\sum_{j}t_{ij}/y_{j}\neq 1$. However, the transposed matrix $A^{⊤}$ is a stochastic matrix, and, by construction, it defines a Markov chain with stationary distribution y, this is, $y=yA^{⊤}$, Equation (13).

In this paper, we make the hypothesis that the Markov chain defined by $A^{⊤}$ is ergodic. First, it has to be irreducible, because all sectors can be reached after some number of transitions, or in other words, all sectors communicate (trade) directly or indirectly after some number of transitions with each other. Second, being irreducible, all states have the same period [23], and it only makes sense that period is 1, this is, the Markov chain is aperiodic, and thus ergodic. Another way to look at irreducibility is to consider a Markov chain as a labeled directed graph where sectors are represented by nodes and edges are given by transitions with nonzero probability [24]. Irreducibility means that the graph is strongly connected, i.e., for each two sectors there is a directed path between them. If SAM were not irreducible, it would mean the existence of sink or drain sectors, which cannot give back to the rest of sectors the money they receive, or source sectors, that can only input money into the other sectors but receive none. In both cases it contradicts the idea of the concept of SAM as a closed (or circular) representation of the economy of a country or region.

The normalized totals of rows or columns y is the equilibrium (or unique stationary) distribution. Starting from any initial distribution $y_{0}$, and with $y_{n}=y_{n-1}A^{⊤}$, $\lim_{n\to \infty }y_{n}=y$. It also means that $\lim_{n\to \infty }{\left(A^{⊤}\right)}^{n}$ is formed by rows equal to y. Observe that $a_{ij}^{⊤}=a_{ji}=t_{ji}/y_{i}$, thus it represents the fraction of total payments from *i* that goes to *j*, or alternatively, the probability that, in a random walk, a unit of money from *i* goes to *j*. If the walk continues infinitely, each state will be visited according to the equilibrium distribution y.

### 3.2. SAM Information Channel

We showed in the previous section that SAM coefficients matrix can be considered an ergodic Markov chain. We interpret this chain here as an information channel.

The elements of a SAM information channel are:

- Being a Markov chain, input and output variables, in our case *X* and *Y*, which represent the economic actors, are equal, and thus probability distributions p(Y) and p(X) are equal to the equilibrium distribution, the normalized *y* vector, y.
- Probability transition matrix $p\left(Y|X\right)=A^{⊤}$ (composed of conditional probabilities $p\left(y|x\right)=a_{ij}^{⊤}$. Each row of SAM matrix, p(Y|X), denoted by $p\left(Y|i\right)=a_{i}^{⊤}$, is a probability distribution.

All these elements are connected by Bayes’ rule that relates marginal (input and output), conditional, and joint probabilities: $p\left(ij\right)=y_{i}a_{ij}^{⊤}=y_{j}a_{ji}^{⊤}$. Thus, the measures of SAM information channel are

- Entropy of the source, $H\left(X\right)=H\left(Y\right)=H\left(y\right)=-\sum_{i}y_{i}\log y_{i}$. Entropy of equilibrium distribution H(y) measures average uncertainty (as an a priori measurement) of input random variable *X*, or alternatively information (as an a posteriori measurement) of output random variable *Y*, both with distribution y. It measures how homogeneous is the importance between the different economic actors. The higher the entropy, the more equal are the actors. A low entropy means that some actors are more important. We can normalize it by the maximum entropy, logM, where *M* is the total number of economic actors.
- Entropy of row *i*, $H\left(Y|i\right)=-\sum_{j}a_{ij}^{⊤}\log a_{ij}^{⊤}$, represents the uncertainty about to which actor *j* will a unit payment from economic actor *i* go. It also measures the homogeneity of the payment flow. If payment from *i* is reduced to a single actor, the entropy of row *i* will be zero, if there is equal payment to all actors the entropy will be maximum. Golan and Vogel [25] consider this entropy, normalized by the maximum entropy logM, as the information of industry *i*.
- Conditional entropy (or entropy of the channel), $H\left(X|Y\right)=H\left(Y|X\right)=H\left(A^{⊤}\right)=\sum_{i}y_{i}H\left(Y|i\right)$. It measures the average uncertainty associated with a payment receptor if we know the emitter. Golan and Vogel [25] consider the non-weighted quantity $\sum_{i}H\left(Y|i\right)$, normalized by MlogM, as reflecting the information in the whole system (*M* industries).
- Mutual information of a row *i*, $I\left(i;Y\right)=\sum_{j}a_{ij}^{⊤}\log\frac{a_{ij}^{⊤}}{y_{j}}$, represents the degree of correlation of economic actor *i* with the rest of the actors. Observe that it is the Kullback-Leibler distance from row *i* to output distribution y. A low value of MI represents a behaviour of payments for actor *i* similar to the distribution y, and that actor *i* behaviour represents the overall behaviour resumed in distribution y. Alternatively, high values of I(i;Y) represent a high deviation from y. This happens for instance if y is very homogeneous, but vector $a_{i}^{⊤}$ has a high inhomogeneity, preferring transitions to a small set of actors, or vice versa, when y is very inhomogeneous and $a_{i}^{⊤}$ is very homogeneous, with similar behaviour with respect to all economic actors.
- Mutual information, $I\left(X;Y\right)=\sum_{i}y_{i}I\left(i;Y\right)$, represents the total correlation, or the shared information, between economic actors, considered to be buyers and providers. We have that $H\left(y\right)=I\left(X;Y\right)+H\left(A^{⊤}\right)$. It is the weighted average of the Kullback-Leibler distances from all rows to input distribution y, where the weight for row *i* is given by $y_{i}$.
- Cross entropy of row i, $CE\left(a_{i}^{⊤},y\right)=H\left(Y|i\right)+I\left(i;Y\right)=-\sum_{j}a_{ij}^{⊤}\log y_{j}$, where $a_{i}^{⊤}$ is the *i* row vector of $A^{⊤}$. Please note that $H\left(y\right)=\sum_{i}y_{i}CE\left(a_{i}^{⊤},y\right)$. Effectively, $H\left(y\right)=I\left(X;Y\right)+H\left(A^{⊤}\right)=\sum_{i}y_{i}H\left(Y|i\right)+y_{i}I\left(i;Y\right)=\sum_{i}y_{i}\left(H\left(Y|i\right)+I\left(i;Y\right)\right)=\sum_{i}y_{i}CE\left(a_{i}^{⊤},y\right)$. As H(y) represents the average uncertainty or information of input and output variables both with distribution y, cross entropy $CE(a_{i}^{⊤},y)$ gives the uncertainty/information associated with actor *i* once we know the channel, which without any knowledge about the channel had been assigned as $-\log y_{i}$.
- Joint entropy, $H\left(X,Y\right)=H\left(X\right)+H\left(Y\right)-I\left(X;Y\right)=2H\left(y\right)-I\left(X;Y\right)=H\left(y\right)+H\left(A^{⊤}\right)$, represents the total uncertainty of the channel. It is the entropy of the joint distribution $p\left(x,y\right)=y_{i}a_{ij}^{⊤}$.

#### 3.2.1. Grouping Sectors

We explained in Section 2.3.1 how to group indexes, and in Equations (14)–(16) the data processing and coarse grain inequalities that hold for the grouping. We explain now in more detail, to the risk of being repetitive, the grouping for SAM, together with the data processing inequality for the mutual information. To group any number of indexes in the SAM matrix $T=t_{ij}$, 1≤i,j≤M, we can do it two at a time, thus we will consider here only grouping of two indexes, and without loss of generality, the last and last but one index. If the grouped matrix is $T^{\prime }$, with elements $t_{ij}^{\prime }=t_{ij}$, 1≤i,j≤M−2, $t_{i,M-1}^{\prime }=t_{i,M-1}+t_{iM}$, 1≤i≤M−2, $t_{M-1,j}^{\prime }=t_{M-1,j}+t_{M,j}$, 1≤j≤M−2, $t_{M-1,M-1}^{\prime }=t_{M-1,M-1}+t_{M-1,M}+t_{M,M-1}+t_{M,M}$, the new totals are $y_{j}^{\prime }=y_{j},1\leq j\leq M-2$, $y_{M-1}^{\prime }=y_{M-1}+y_{M}$, which by construction are the same summing by row than by column, thus defining $A^{\prime }$ as $a_{ij}^{\prime }=t_{ij}^{\prime }/y_{j}^{\prime }$ we have that $y^{\prime }=A^{\prime }y^{\prime }$. This is, the grouped totals are the equilibrium distribution of the new SAM coefficient matrix obtained by grouping the original *T* matrix into $T^{\prime }$. Observe now that $\sum_{i,j=1}^{M}t_{ij}=\sum_{i,j=1}^{M}t_{ij}^{\prime }=t$, and the distribution $T^{\prime }/t={\left\{t_{ij}^{\prime }/t\right\}}_{i,j=1}^{M}$ is a grouping of the distribution $T/t={\left\{t_{ij}/t\right\}}_{i,j=1}^{M}$, and thus by the data processing inequality $D_{KL}\left(T^{\prime }/t,\left\{{y^{\prime }}_{i}{y^{\prime }}_{j}\right\}\right)\leq D_{KL}\left(T/t,\left\{y_{i}y_{j}\right\}\right)$, which by Equations (7)–(9) is equivalent to the decrease in mutual information when grouping.

From now on, and to avoid cluttering of notation, we will drop the transpose symbol from the $A^{⊤}$ matrix, and we will simply refer to it as *A* matrix.

## 4. Cross Entropy Method

By balancing a SAM coefficient matrix it is understood in the literature to obtain the values of a SAM coefficients matrix, *A*, where we know only the totals and a previous SAM matrix, $\bar{A}$, which is the a priori knowledge. The state of the art balancing methods are based in minimizing information theoretic quantities. In this section, we revise the different objective balancing functions, investigate the relationship between them, prove that the proposed functions are 0 if and only if $A\equiv \bar{A}$, and show its relationship to the channel quantities defined in Section 3.2.

A main problem in constructing the SAM matrix is that we often only have partial information. The cross entropy method introduced by Golan et al. [11,26] to update a SAM coefficient matrix $\bar{A}$, with equilibrium distribution $\bar{y}$, to a new partially unknown stochastic matrix *A* from which we know the equilibrium distribution y (subjected thus to condition y = y A), consists of completing matrix *A* through minimizing the following expression

(17) $$I_{1}=\sum_{i}\sum_{j}a_{ij}\log\frac{a_{ij}}{{\bar{a}}_{ij}},$$

where $I_{1}$ is the sum of Kullback-Leibler distances between the rows of partially unknown *A* and the rows of known $\bar{A}$. See also extensions by Golan and Vogel [25] and a more recent summary by Robinson et al. [27]. As a Kullback-Leibler distance is always positive, we have that $I_{1}\geq 0$, being equal to 0 only when for all i,j, $a_{ij}={\bar{a}}_{ij}$, that is $A\equiv \bar{A}$. Please note that in $I_{1}$ we do not take into account the weight $y_{i}$ of each row. If we take it into account we can define the objective function [28]

(18) $$I_{1}^{\prime }=\sum_{i}y_{i}\sum_{j}a_{ij}\log\frac{a_{ij}}{{\bar{a}}_{ij}},$$

subjected to the same constraints as before. $I_{1}^{\prime }$ is always positive as is the average sum of Kullback-Leibler distances, and as $I_{1}$, only equal to 0 when $A\equiv \bar{A}$ (we suppose $y_{i}>0$ for all *i*). $I_{1}^{\prime }$ can be written in the form

(19) $$\begin{array}{ccc} I_{1}^{\prime } & = & \sum_{i}y_{i}\sum_{j}a_{ij}\log a_{ij}-\sum_{i}y_{i}\sum_{j}a_{ij}\log{\bar{a}}_{ij}\\ & = & -\sum_{i}y_{i}H\left(a_{i}\right)+\sum_{i}y_{i}CE\left(a_{i},{\bar{a}}_{i}\right)\\ & = & -H\left(X|Y\right)+\sum_{i}y_{i}CE\left(a_{i},{\bar{a}}_{i}\right) \end{array}$$

Observe that for the particular case where for all *i* the row vectors ${\bar{a}}_{i}=y_{i}$ then $\sum_{i}y_{i}CE\left(a_{i},{\bar{a}}_{i}\right)=\sum_{i}y_{i}CE\left(a_{i},y_{i}\right)=H\left(y\right)$ (see Section 3.2), and $I_{1}^{\prime }$ becomes I(X;Y).

McDougall [28] also defined the objective function

(20) $$I_{2}=\sum_{i}\sum_{j}y_{i}a_{ij}\log\frac{y_{i}a_{ij}}{{\bar{y}}_{i}{\bar{a}}_{ij}},$$

where $I_{2}$ is the Kullback-Leibler distance between the new and the a priori joint distributions, given by $y_{i}a_{ij}$ and ${\bar{y}}_{i}{\bar{a}}_{ij}$ respectively ($\sum_{ij}y_{i}a_{ij}=\sum_{ij}{\bar{y}}_{i}{\bar{a}}_{ij}=1$). Being a Kullback-Leibler distance, it is always positive, and only equal to 0 when for all i,j, $y_{i}a_{ij}={\bar{y}}_{i}{\bar{a}}_{ij}$. Observe that it does not directly imply that for all i,j, $a_{ij}={\bar{a}}_{ij}$, although we will see below that this is the case.

A lower bound of $I_{2}$ can be obtained with the log-sum inequality [9],

(21) $$\begin{array}{ccc} I_{2} & = & \sum_{i}\sum_{j}y_{i}a_{ij}\log\frac{y_{i}a_{ij}}{{\bar{y}}_{i}{\bar{a}}_{ij}}\geq \sum_{i}\left(\sum_{j}y_{i}a_{ij}\right)\log\frac{\left(\sum_{j}y_{i}a_{ij}\right)}{\left(\sum_{j}{\bar{y}}_{i}{\bar{a}}_{ij}\right)}=\sum_{i}y_{i}\log\frac{y_{i}}{{\bar{y}}_{i}}, \end{array}$$

which being a Kullback-Leibler distance is always positive and only equal to zero when $y=\bar{y}$. Thus, $I_{2}$ is 0 *iff* for all *i*, $y_{i}={\bar{y}}_{i}$, and *iff* for all i,j, $y_{i}a_{ij}={\bar{y}}_{i}{\bar{a}}_{ij}$. Thus, $I_{2}$ is 0 *iff* for all i,j, $a_{ij}={\bar{a}}_{ij}$, that is $A\equiv \bar{A}$.

Proceeding as with $I_{1}^{\prime }$, $I_{2}$ can be written as

(22) $$I_{2}=-H\left(X,Y\right)+CE\left(y_{i}a_{ij},{\bar{y}}_{i}{\bar{a}}_{ij}\right).$$

The function $I_{2}$ is more directly related to the monetary flow, as $y_{i}a_{ij}=y_{i}/t\times t_{ij}/y_{i}=t_{ij}/t$, where $t=\sum_{i}y_{i}=\sum_{i,j}t_{ij}$, and then $I_{2}$ can be written as

(23) $$I_{2}=\sum_{i}\sum_{j}t_{ij}/t\log\frac{t_{ij}/t}{{\bar{t}}_{ij}/\bar{t}}=\frac{1}{t}\sum_{i}\sum_{j}t_{ij}\log\frac{t_{ij}}{{\bar{t}}_{ij}}+\log\frac{\bar{t}}{t}$$

subject to restriction for all *i*, $y_{i}=\sum_{j}t_{ij}=\sum_{j}t_{ji}$.

Observe that, being *t* and $\bar{t}$ constant, minimizing $I_{2}$ is the same that minimizing the following quantity

(24) $$I_{2}^{\prime }=\sum_{i}\sum_{j}t_{ij}\log\frac{t_{ij}}{{\bar{t}}_{ij}}.$$

Although $I_{2}\geq 0$ because it is a Kullback-Leibler distance, $I_{2}^{\prime }$ can be negative as the $t_{ij}$ values are not normalized. We can bound $I_{2}^{\prime }$ from below using the log-sum inequality,

(25) $$\begin{array}{ccc} I_{2}^{\prime } & = & \sum_{i}\sum_{j}t_{ij}\log\frac{t_{ij}}{{\bar{t}}_{ij}}\geq \left(\sum_{i}\sum_{j}t_{ij}\right)\log\frac{\left(\sum_{i}\sum_{j}t_{ij}\right)}{\left(\sum_{i}\sum_{j}{\bar{t}}_{ij}\right)}=t\log\frac{t}{\bar{t}}, \end{array}$$

with equalities only when for all i,j, $t_{ij}=c{\bar{t}}_{ij}$. However, $t=\sum_{i,j}t_{ij}=c\sum_{i,j}{\bar{t}}_{ij}=c\bar{t}$, thus $c=\frac{t}{\bar{t}}$. As $a_{ij}=\frac{t_{ij}}{\sum_{j}t_{ij}}$ and ${\bar{a}}_{ij}=\frac{{\bar{t}}_{ij}}{\sum_{j}{\bar{t}}_{ij}}$, equality in Equation (25) happens *iff* for all i,j, $a_{ij}={\bar{a}}_{ij}$, that is $A\equiv \bar{A}$.

McDougall [28] stated that minimizing $I_{1}^{\prime }$ was equivalent to minimizing $I_{2}$, and proved that the minimum of $I_{2}$ was the RAS solution, $t_{ij}=r_{i}{\bar{t}}_{ij}s_{j}$, where $r_{i}$ and $s_{i}$ are scaling row and column factors, respectively. We give now a proof of the equivalence of minimizing $I_{1}^{\prime }$ and $I_{2}$. We have that

(26) $$\begin{array}{ccc} I_{2}-I_{1}^{\prime } & = & \sum_{i}y_{i}\left(\sum_{j}a_{ij}\log\frac{y_{i}a_{ij}}{{\bar{y}}_{i}{\bar{a}}_{ij}}-\sum_{j}a_{ij}\log\frac{a_{ij}}{{\bar{a}}_{ij}}\right)\\ & = & \sum_{i}y_{i}\left(\sum_{j}a_{ij}\left(\log\frac{y_{i}}{{\bar{y}}_{i}}+\log\frac{a_{ij}}{{\bar{a}}_{ij}}\right)-\sum_{j}a_{ij}\log\frac{a_{ij}}{{\bar{a}}_{ij}}\right)\\ & = & \sum_{i}y_{i}\sum_{j}a_{ij}\log\frac{y_{i}}{{\bar{y}}_{i}}=\sum_{i}y_{i}\log\frac{y_{i}}{{\bar{y}}_{i}} \end{array}$$

which is the Kullback-Leibler distance between the new and old row totals. As vectors $y,\bar{y}$ are known a priori, minimizing $I_{2}$ is equivalent to minimizing $I_{1}^{\prime }$.

We can play with the relationship between channel quantities to obtain new optimization functions. For instance, adding −I(X;Y) to Equation (19) we obtain

(27) $$\begin{array}{ccc} I_{1}^{\prime \prime } & = & -I\left(X;Y\right)-H\left(X|Y\right)+\sum_{i}y_{i}CE\left(a_{i},{\bar{a}}_{i}\right)\\ & = & -H\left(X\right)+\sum_{i}y_{i}CE\left(a_{i},{\bar{a}}_{i}\right), \end{array}$$

which is equivalent to optimizing $\sum_{i}y_{i}CE\left(a_{i},{\bar{a}}_{i}\right)$, as $H\left(X\right)=-\sum_{i}y_{i}\log y_{i}$ is known a priori. In the same way, adding H(X|Y) to Equation (22) we can define

(28) $$I_{2}^{\prime \prime }=H\left(X|Y\right)-H\left(X,Y\right)+CE\left(y_{i}a_{ij},{\bar{y}}_{i}{\bar{a}}_{ij}\right)=-H\left(X\right)+CE\left(y_{i}a_{ij},{\bar{y}}_{i}{\bar{a}}_{ij}\right),$$

which is equivalent to optimizing $CE(y_{i}a_{ij},{\bar{y}}_{i}{\bar{a}}_{ij})$ as H(X) is known a priori.

## 5. Examples

We show in this section examples for Austria SAM 2010 matrix and South Africa SAM time series matrixes 1993–2013. Please note that as we work with the transpose of the original *A* matrix, when we talk about entropy or mutual information of row we refer to the corresponding column in matrix *A*.

### 5.1. Austria SAM 2010 Matrix

**Figure 3:**
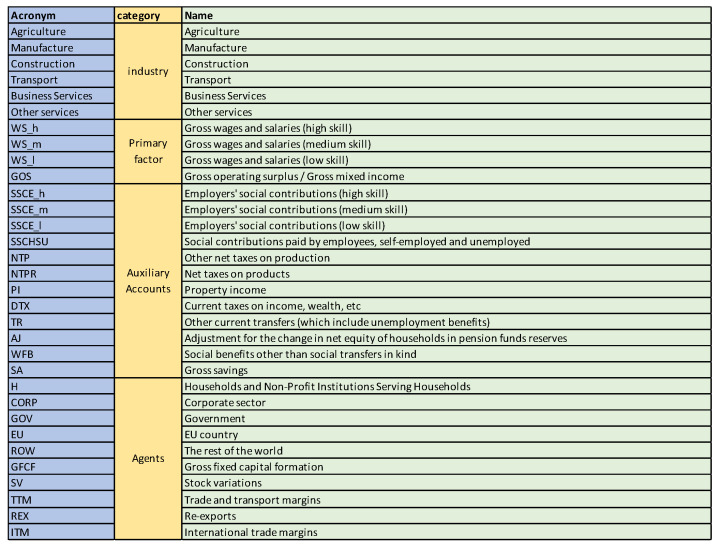
The sectors for Austria 2010 SAM.

**Figure 4:**
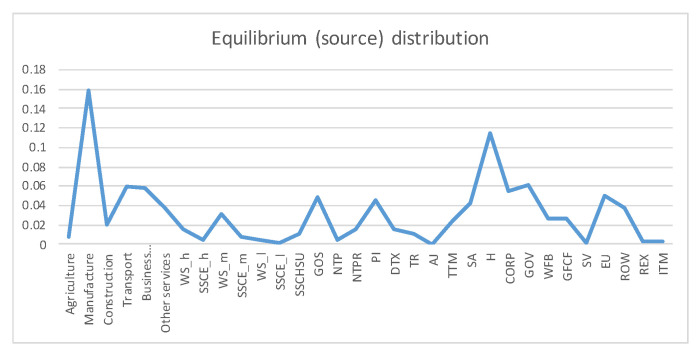
The equilibrium distribution y for Austria 2010 SAM. The horizontal axis represents the sectors, their description is in Figure 3. The vertical axis gives the relative frequency, or weight, of each sector. The two more important sectors are Manufacture and Household. The inhomogeneity between sectors is measured by the entropy of the source, shown in Table 1.

**Figure 5:**
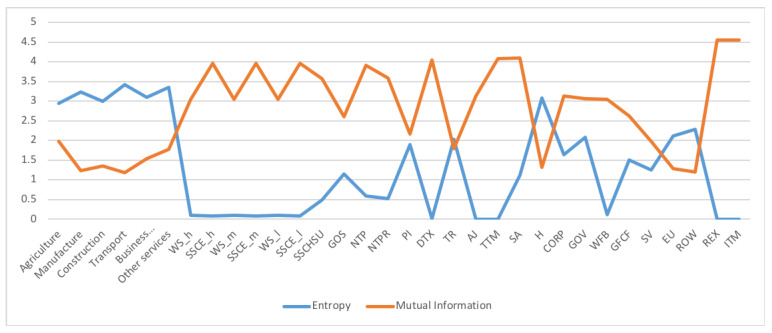
The row mutual information and row entropy for Austria 2010 SAM. The horizontal axis represents the sectors, their description is in Figure 3. Observe that both quantities take almost complementary values. The sectors with higher mutual information (and lower entropy) are strongly connected to a few other sectors, while sectors with higher entropy (and lower mutual information) are connected more homogeneously with more sectors. Observe that for the first six sectors, and Households and EU sector, the entropy is higher than the mutual information.

A first example is the analysis of SAM 2010 matrix for Austria, data obtained from [29]. The SAM matrix contains 32 sectors, see Figure 3. We checked that the SAM coefficient matrix *A* corresponds to an ergodic Markov chain, by taking the powers $A^{n}$. For n>20 the rows are practically equal to the stationary distribution given by the normalized totals of the rows, and thus the stationary distribution is unique and is the equilibrium distribution, which acts as source to the channel. Then we computed the quantities of the information channel, see second column of Table 1. The entropy of the source is 4.290 (out of a maximum possible entropy of $\log_{2}32=5$), which splits into the entropy of the channel, 2.136, and the mutual information. From the high value of the entropy we can deduce a relatively homogeneous distribution between sectors, see Figure 4. The equilibrium distribution has some spikes at Manufacture and Household sectors. We observe also that the relationship of the entropy of channel value to the mutual information value is practically equal to 1. In terms of channel interpretation, we could say that both randomness and determinism take equal share on average. It might be a characteristic of a developed market. If we consider the SAM coefficient matrix as describing an economics complex system, for the effective complexity to be sizable, the system must be neither too orderly nor too disorderly [30]. We can see in Figure 5 the sector by sector distribution of entropy and mutual information. A high entropy (and thus a low mutual information) would mean a highly distributed output from the sector. A high mutual information (and thus a low entropy) would mean a highly focalized output from the sector. From Figure 5 we see that only in half a dozen sectors entropy and mutual information are equal, while in the other sectors either one or the other predominate.

### 5.2. Dual channel for Austria SAM 2010

**Figure 6:**
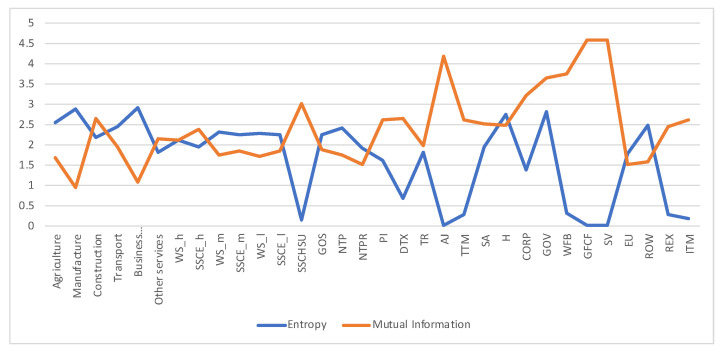
The row mutual information and row entropy for Austria 2010 SAM dual channel, compare with Figure 5. The horizontal axis represents the sectors, their description is in Figure 3. Entropy and mutual information are in general more balanced than in Figure 5.

**Figure 7:**
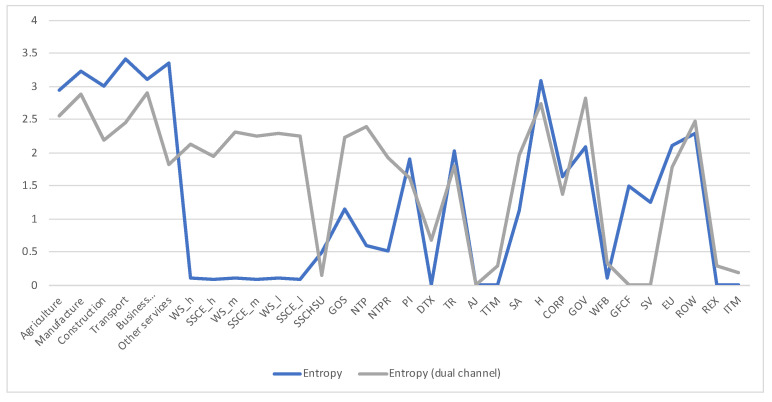
The row entropy for Austria 2010 SAM channel (representing supply or payments made) and dual channel (representing demand or payments received) compared. The horizontal axis represents the sectors, their description is in Figure 3. The main differences are in industry sectors, primary factor and corresponding social contributions, taxes, government, capital, stock and EU. Looking at the original data from [29], we find that almost all payments made by wages sectors go to just one sector, Households, and social contributions just to Government, thus the very low entropy, while almost all payments received by wages and social contributions sectors come from several sectors, the industry ones, thus their higher entropy. Industry sectors present also less entropy in the dual channel, it means that the diversification of demand is lower than the supply one, industry sectors made payments up to 20 sectors, while received payments from up to 12 sectors. The lower entropy in Government sector for the dual channel means that payments by the Government sector are concentrated in fewer sectors than the payments received. Gross fixed capital formation sector receives payment just from one sector, Gross savings, hence the zero entropy in dual channel, while it contributes to all industry sectors, hence the non-zero entropy. Similarly, Stock variations is only contributed by Gross savings, while it contributes to most of the industries. Current taxes on income sector pays practically only to Government sector, hence entropy practically zero, while it receives payments mainly from Households and Corporations.

Remember that the channel discussed so far with transition matrix $A^{⊤}$ has been obtained by normalizing the columns of the total payements *T* matrix. Following Section 2.3.2 we can define the *dual* channel ${\left(A^{⊤}\right)}^{d}$, which will be obtained by normalizing rows. Channel $A^{⊤}$ represents the payements made (or supply), and dual channel ${\left(A^{⊤}\right)}^{d}$ the payements received (or demand). As discussed in Section 2.3.2 the equilibrium distribution and the measures H(X),H(X,Y),H(Y|X),I(X;Y) are the same in both channels, but the row entropy and row mutual information are not. We show in Figure 6 the values for the dual channel. We compare in Figure 7 the row entropies for the two channels, the row entropy of $A^{⊤}$ channel tells us how diversified are the suppliers, while the row entropy of the dual channel $A^{⊤}$ tells us how diversified is the demand.

#### 5.2.1. Examining the Role of the Data Processing Inequality in Grouping

To illustrate about the role that data processing inequality can play in the grouping of sectors in a SAM matrix, we consider now the variation in mutual information if we group some columns (and corresponding rows) in the 2010 SAM Austria matrix. According to Section 3.2.1 the grouping will leave the equilibrium distribution invariant except for the indexes that are grouped, that will be substituted by their sum. We will group alternatively high salary and middle salary, middle salary and low salary, and high, middle and low salary together, and the same with the respective employers’ social contributions. The results are presented in Table 1. The second column in Table 1 corresponds to the original SAM matrix, with 32 rows (columns), the third column in Table 1 to the grouping of high salary and middle salary and the respective social contributions, with 30 rows (columns), the fourth column in Table 1 to the grouping of middle salary and low salary and the respective social contributions, also with 30 rows (columns), and the last column in Table 1 to the grouping of high, middle and low salaries and respective social contributions, with 28 rows (columns). As expected from the data processing inequality (Equation (10)) and the coarse grain property (Equation (3)) when grouping, the values of the entropies of the source, the total entropy, and the mutual information decrease in third, fourth and last column (see Equation (14)). Observe that we could consider the reverse, that is, start from a 28 row (column) SAM matrix with a single row for salary and employer’s contribution level and refine it, the data processing inequality tells us that there will be an increment in 30 and 32 rows SAM matrices with respect to the 28 ones. We observe that the lesser difference happens when we group middle and lower salary levels. Bottleneck method [31] is based on grouping according to the less decrease of mutual information, but we could use the less decrease, by coarse grain property, in entropy of the source or the total entropy. In our example the three cases happen to result in recommending the same grouping, but it has not to be so in general.

### 5.3. South Africa SAM Time Series Matrixes 1993–2013

**Figure 8:**
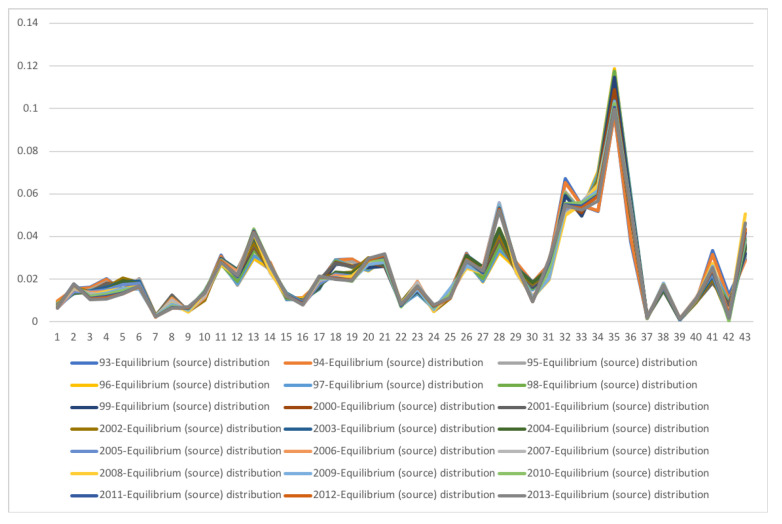
The equilibrium distribution y for SA SAM time series (1993–2013) with current prices. Each line corresponds to a year (see the legend in the graph). The horizontal axis represents the sectors, their description is in Figure 11. The vertical axis gives the relative frequency, or weight, of each sector. The sector with highest weight is the 35, Households. The inhomogeneity between sectors is measured by the entropy of the source, shown in Figure 9 for each year. Observe that the shape of distributions is relatively stable over the years, changing little except for certain sectors, which traduces into an almost constant entropy.

**Figure 9:**
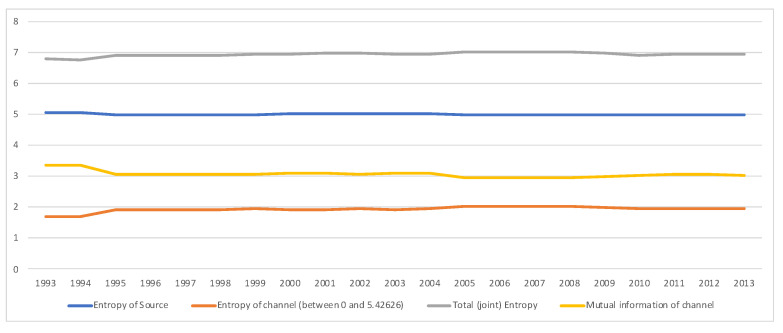
Time series (1993–2013) of the total entropy (in gray), entropy of the source (in blue), entropy of the channel (in red) and mutual information (in yellow) for SA SAM with current prices. Observe that the entropy of the source (entropy of equilibrium distribution y) changes very little in the series. However, the entropy of the channel and the mutual information (which added are equal to the entropy of the source) tend to get closer. See a close-up of entropy of the channel and mutual information time series in Figure 12.

**Figure 10:**
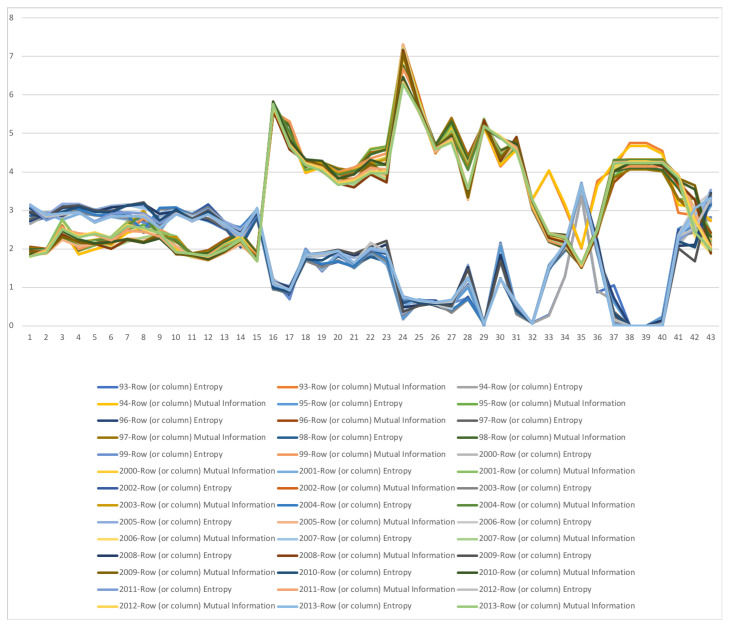
The row mutual information (warm colors) and entropy (cold colors) for SA SAM time series (1993–2013) with current prices. The horizontal axis represents the sectors, their description is in Figure 11. Each line corresponds to a year (see the legend in the graph). Observe the change in the values for sectors 33–36 (Capital, Enterprises, Households, Government), denoting a change, or restructuring, in the connections of these sectors with the other sectors. Observe also that some sectors have higher entropy and other sectors higher mutual information. Sectors with highest mutual information are sectors 16 (Agriculture) and 24 (Electricity, Gas and water supply), meaning that they have strong connections with a few sectors, while the sector with higher entropy is sector 35 (Households), meaning it is connected with many sectors and in a more even way. Observe the same behaviour for Households than in Austria SAM in Figure 5.

**Figure 11:**
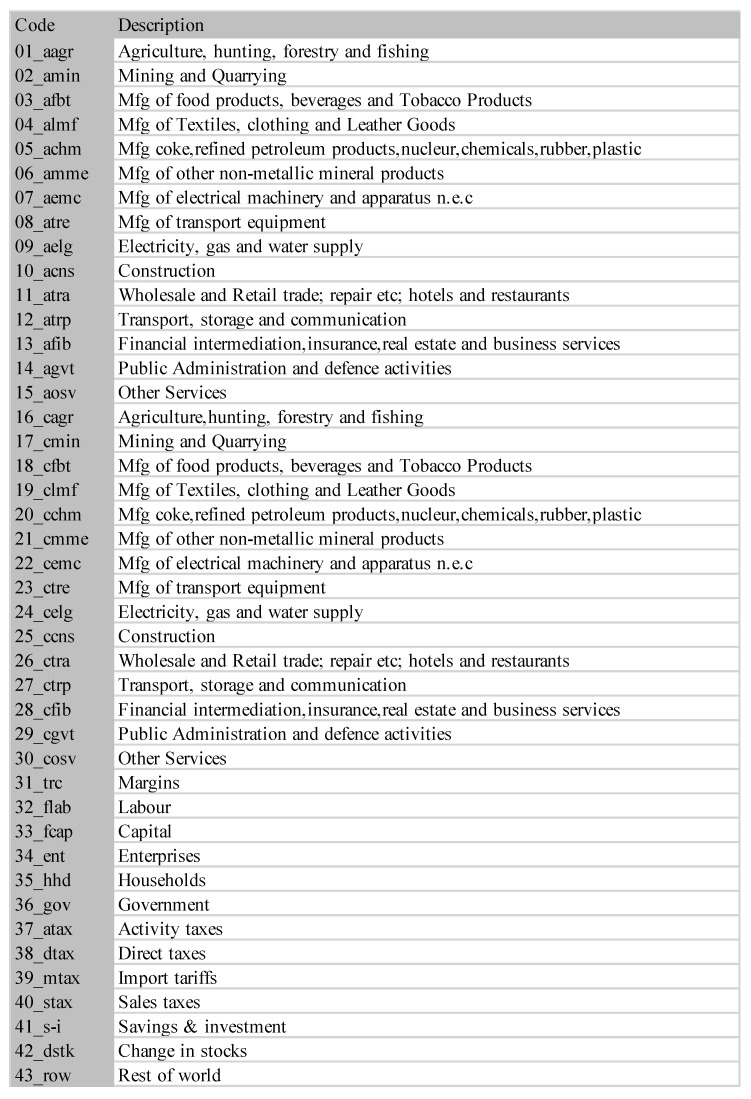
The sectors for SA SAM time series (1993–2013). Sectors 1–15 correspond to activities and 16–30 to commodities.

**Figure 12:**
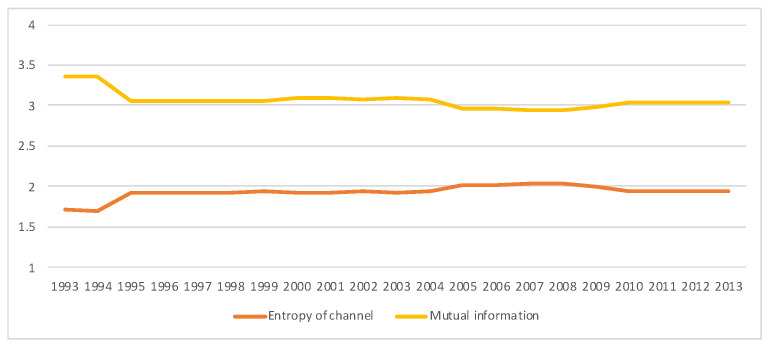
Close-up from Figure 9, illustrating the variations of entropy of the channel and mutual information for SA SAM time series (1993–2013) with current prices. An interpretation of this evolution is that in a developing economy, mutual information and entropy of channel tend to become relatively equal (as in Austria SAM 2010 case), with significant changes in 1995 and 2005, being the process interrupted in 2008, with a significant change in 2010.

**Figure 13:**
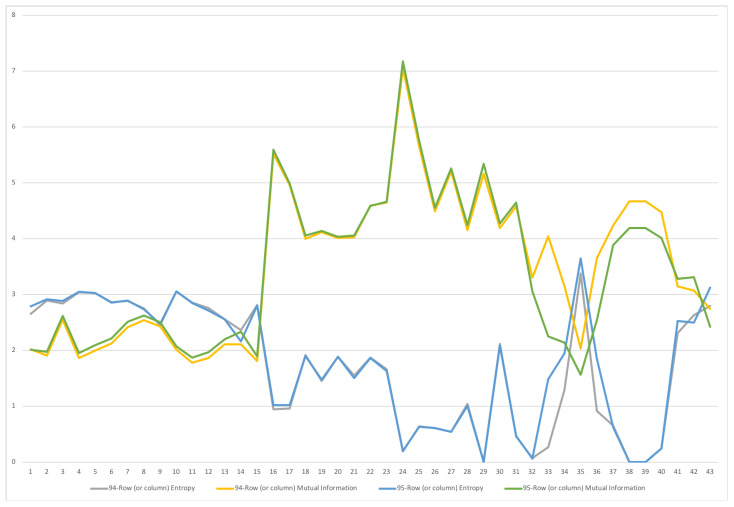
The row mutual information (warm colors) and entropy (cold colors) for SA SAM time series with current prices for 1994–1995. The horizontal axis represents the sectors, their description is in Figure 11. Each line corresponds to a year (see the legend in the graph). Important changes happen in sectors 33 through 42, from Capital to Change in stocks. There is also a small increase in row entropy in all activities sectors, 1–15. This might be due to either a small error or bias in balancing the SAM, or a small change in the equilibrium distribution, as mutual information of a row is the K-L distance to equilibrium distribution. A change in supply seems not under consideration as row entropies do not change for these sectors.

**Figure 14:**
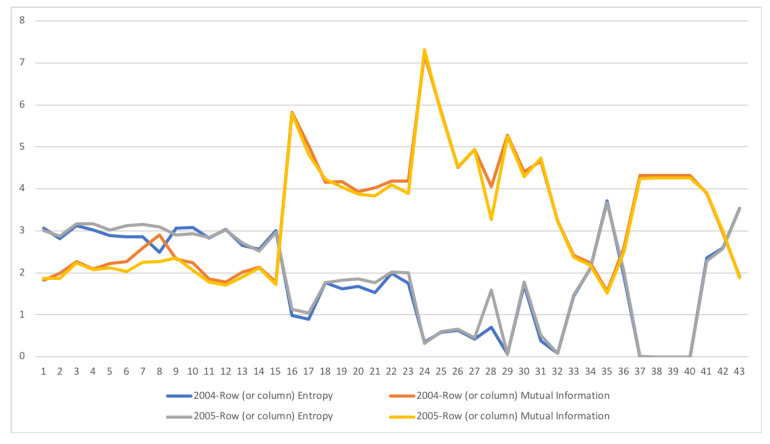
The row mutual information (warm colors) and entropy (cold colors) for SA SAM time series with current prices for 2004–2005. The horizontal axis represent the sectors, their description is in Figure 11. Each line corresponds to a year (see the legend in the graph). Changes only happen in activities (1–15) sectors and their corresponding commodities (16–30). Main changes happen in Manufacturing (3–8, 19–23) sectors, both in supply and demand, and in Financial intermediation, only in demand (28). In this last sector, entropy increased and mutual information decreased, meaning that Financial intermediation demand sector related to more sectors and in a more homogeneous way.

**Figure 15:**
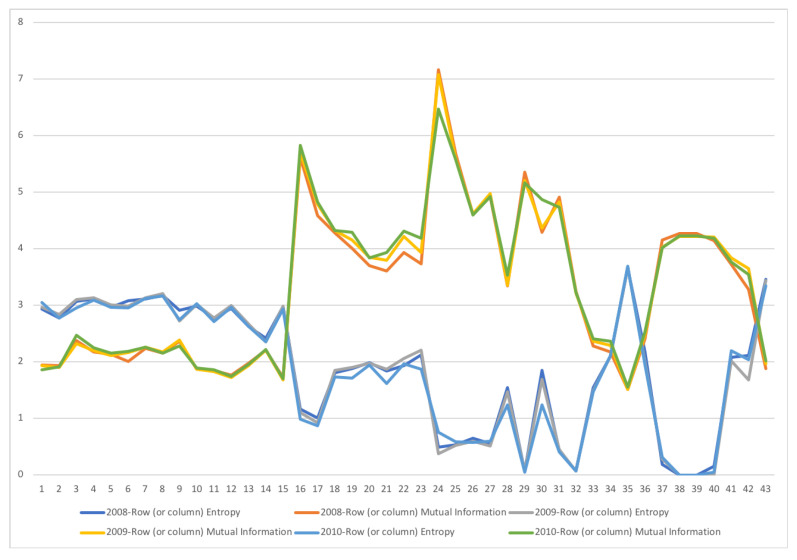
The row mutual information (warm colors) and entropy (cold colors) for SA SAM time series with current prices for 2008–2010. The horizontal axis represents the sectors, their description is in Figure 11. Each line corresponds to a year (see the legend in the graph). There is change in many sectors of the economy, although the most important changes happen in demand in sector 30, Other services, and sector 42, Change in stock. Other sectors that have noticeable changes are demand Manufacture sectors, from 19 to 22, where demand is more concentrated.

**Figure 16:**
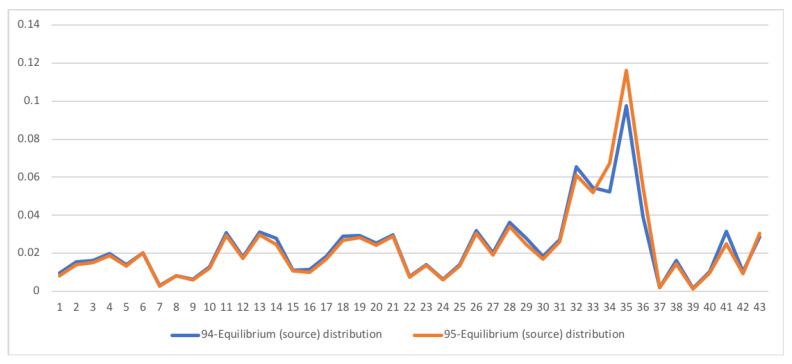
The equilibrium distribution y for SA SAM time series with current prices for 1994-1995. Each line corresponds to a year (see the legend in the graph). The horizontal axis represents the sectors, their description is in Figure 11. Enterprises and Households sectors (sectors 34 and 35) increase their weight while Savings sector (sector 41) decreases.

**Figure 17:**
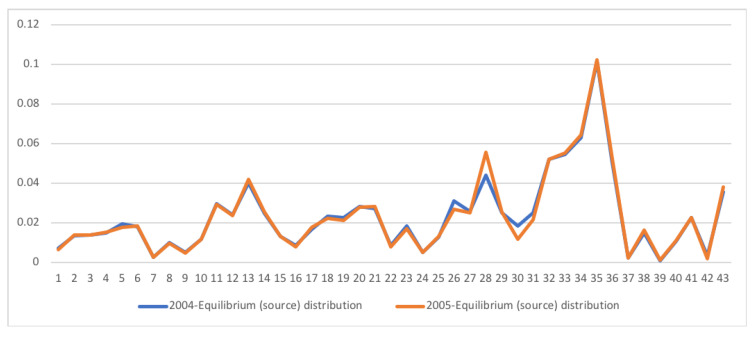
The equilibrium distribution y for SA SAM time series with current prices for 2004–2005. Each line corresponds to a year (see the legend in the graph). The horizontal axis represents the sectors, their description is in Figure 11. Demand sectors Wholesale and Other services (sectors 26 and 30) decrease their weight while demand sector Financial intermediation (sector 28) increases.

**Figure 18:**
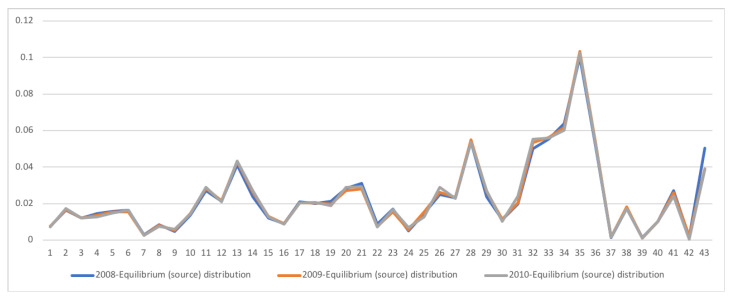
The equilibrium distribution y for SA SAM time series with current prices for 2008–2010. Each line corresponds to a year (see the legend in the graph). The horizontal axis represents the sectors, their description is in Figure 11. The most noticeable change is the decrease of sector Rest of World (sector 43), and a small increase in Labour and Wholesale sectors (sectors 32 and 26).

A second example is the temporal series of SAM for current prices for South Africa between years 1993 and 2013. The data has been obtained from [32]. The equilibrium distribution for the time series is shown in Figure 8. The total entropy, entropy of the source, entropy of the channel, and mutual information of the time series can be shown in Figure 9. The entropy and mutual information for each sector are shown in Figure 10, where the coding of each sector is given in Figure 11. Each line in Figure 10 represents one single year. From Figure 9 we can observe that the entropy of the source is relatively stable along the years, with a little decrease in the first three years. This stability makes the behaviour of the entropy of the channel and of the mutual information mirror each other, that is, the decrease of mutual information is compensated by an increase in channel entropy, thus we will discuss only mutual information (MI) here. Observe also that MI is higher than the entropy of the channel. MI has an important decrease from 1995 to 1998, and then decreases slowly till 2008, with a steep decrease in 2005. We zoom on its behavior in Figure 12. From 2008 it increases again. This is coincident with the financial global crisis of 2008. It might be that a developing market has a higher MI than channel entropy, and it tries to balance both quantities in the development process. A higher MI means that output of a sector is directed to just a few other sectors, thus a market would be formed by a kind of small scope circuits put together. When channel entropy increases, it would mean that those circuits are reaching more sectors. It might be that the balance is the one seen in the example above about Austria 2010 SAM. From Figure 10 we detected that main changes happened in 1994–1995, 2004–2005, and 2008–2010, thus we plot in Figure 13, Figure 14 and Figure 15 the changes in 1994–1995, 2004–2005 and 2008–2010 respectively so that the sectors where the changes happen can be identified. We also plot in Figure 16, Figure 17 and Figure 18 the equilibrium distributions of 1994–1995, 2004–2005 and 2008–2010 respectively. We give in the captions of Figure 13, Figure 14 and Figure 15 possible explanations for those changes, although some caution has to be taken, because as data is fully updated only every 5 years, and interpolated (or balanced) in the in-between years, it might be for instance that changes that appear only from 1994 to 1995 might correspond to changes over a five year period.

#### Grouping Sectors

As in Section 5.2.1 we examine now for SA 2013 current price SAM matrix the change in the several quantities for grouping different sectors. The results are presented in Table 2. In the second column of Table 2 we have the results of the original 43 rows matrix. In the further columns we present different groupings, by aggregating affine sectors. Observe that whenever we aggregate, by the data processing inequality for mutual information and the coarse grain property for entropies, these values decrease for the grouped matrices. We could choose which sectors to aggregate according to the minimum decrease in mutual information. In addition, observe that aggregating activities sectors 1 (Agriculture) and 3 (Manufacturing of food products), and the corresponding commodities sectors 16 and 18, and activities sectors 2 (Mining), 5 (Manufacturing of coke, refined petroleum products...) and 9 (Electricity, gas and water supply) and the corresponding commodities sectors 17, 20 and 24 at the same time, the values for mutual information and for entropy of source and joint entropy are less than for just aggregating 1 and 3 (and 16 and 18) sectors, 2 and 5 (and 17 and 20), 5 and 9 (and 20 and 24), 2 and 9 (and 17 and 24), and 2, 5 and 9 (and 17, 20 and 24), as it should be, because aggregating 1 and 3 (and 16 and 18) sectors and 2,5 and 9 (and 17, 20 and 24) is a grouping of any of those possibilities.

## 6. Conclusions

We showed first that a SAM coefficient matrix can be interpreted as an ergodic Markov chain, and then extended it as an information channel. We saw that this interpretation as an information channel is fully compatible with the cross entropy and related methods used to obtain missing information to build up the SAM matrix, and shown the relationship between the different objective functions themselves and with the channel quantities. We presented several examples of SAM information channels, computing the different quantities of the channel, as the entropy of the source, the entropy of the channel, and the mutual information and entropy of each row, and given an interpretation to each of these quantities. We also explored the grouping of sectors in the context of data processing inequality.

In the future, we will consider extending our framework from Shannon entropy to Rényi entropy [33], and compare these entropies with other diversity indexes in the literature [14] (in fact, Rényi entropy with parameter equal to 2 is directly related to Herfindahl-Hirschman diversity index). We will also explore the input output matrices as information channels, as they have been already interpreted as Markov chains, see for instance [34]. Although the examples presented in this paper corresponded to ergodic Markov chains, ergodicity is not necessary to interpret a Markov chain as an information channel, something that will be useful when dealing with an absorbing Markov chain as in [35]. 

## Figures and Tables

**Table 1:** The channel quantities values for original Austria 2010 SAM in the second column. Observe that mutual information and entropy of channel are practically equal. In the third row we grouped Gross wages and salaries (high skill) with Gross wages and salaries (medium skill), and Employers’ social contributions (high skill) with Employers’ social contributions (medium skill). In the fourth columns we grouped Gross wages and salaries (medium skill) with Gross wages and salaries (low skill), and Employers’ social contributions (medium skill) with Employers’ social contributions (low skill). In the fifth and last column we grouped all three salary and social contribution levels. Observe that all quantities decrease when grouping (even entropy of channel which does not hold the data processing inequality), and minimum decrease happens when grouping medium and low levels.

Grouped Sectors (Wages and Social Contributions)	No Grouping	High + Medium	Medium + Low	High + Medium + Low
Entropy of source H(X)	4.29052	4.23750	4.26602	4.20983
Conditional entropy H(X|Y)	2.13603	2.08578	2.11160	2.05848
Total (Joint) entropy H(X,Y)	6.42656	6.32328	6.37762	6.26831
Mutual information I(X;Y)	2.15449	2.15171	2.15442	2.15135

**Table 2:** The channel quantities values for SA 2013 SAM with current values, with the original 43 rows in the second column, and several alternative grouping of different sectors in the further columns. The sectors are (from description in Figure 11) 1 and 16 (Agriculture), 2 and 17 (Mining), 3 and 18 (Manufacturing of food products), 5 and 20 (Manufacturing of coke, refined petroleum products, nuclear, chemical and rubber plastic) and 9 and 24 (Electricity, gas and water supply). Observe that all quantities (even in this case entropy of channel, which does not hold the data processing inequality) decrease when grouping.

Grouped Sectors	No Grouping	1-3, 16-18	2-5,17-20	2-9, 17-24	5-9, 20-24	2-5-9, 17-20-24	1-3, 2-5-9, 16-18, 17-20-24
Entropy of source	4.98261	4.94231	4.90204	4.93709	4.93629	4.84288	4.80258
Entropy of channel	1.9502	1.9343	1.93281	1.94083	1.93744	1.90876	1.89281
Total (Joint) entropy	6.93282	6.87661	6.83484	6.87792	6.87373	6.75165	6.69539
Mutual information	3.03241	3.00801	2.96923	2.99626	2.99885	2.93412	2.90977

**Figure A1:**
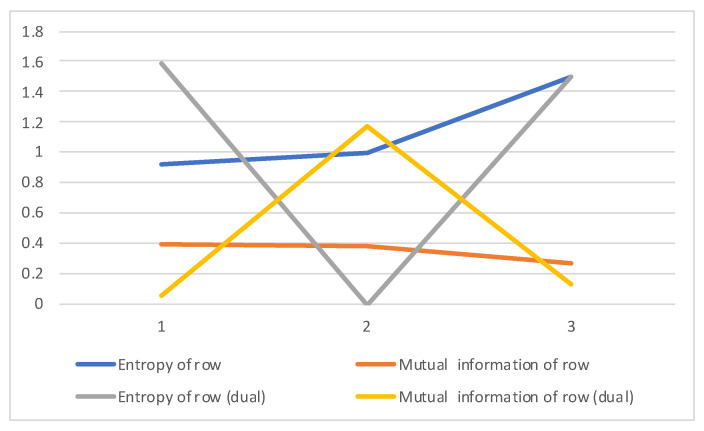
The entropies and mutual informations of both *A* and its dual $A^{d}$ channels for our toy example (values are in Table A2). Entropies and mutual informations of *A* channel do not show much variation, as payments are made relatively homogeneously to at least two out of the three sectors (see the rows in matrix *A*). As for the $A^{d}$ channel, we observe that sector 2 has zero entropy, as it pays only to one sector, and for the same reason has maximum mutual information, while sector 1 has maximum entropy, and minimum mutual information, as it makes equal payments to all three sectors (see the rows in matrix $A^{d}$).

## Appendix A. A SAM Toy Example

### Appendix A.1. Ergodic Markov Chain

Let us suppose a 3 × 3 SAM matrix *T* where sums of rows and columns are equal: $\left(\begin{array}{ccc} 1 & 1 & 1\\ 0 & 0 & 2\\ 2 & 1 & 1 \end{array}\right)$.

The total sum of the elements of the matrix *T* is t=9.

The row/column totals are y={3,2,4}.

The normalized row/column totals are y={1/3,2/9,4/9}.

If we normalize the columns of matrix *T* by the column totals and transpose we obtain matrix *A*:

$\left(\begin{array}{ccc} \frac{1}{3} & 0 & \frac{2}{3}\\ \frac{1}{2} & 0 & \frac{1}{2}\\ \frac{1}{4} & \frac{1}{2} & \frac{1}{4} \end{array}\right)$. If we normalize the rows of matrix *T* by the row totals we obtain the dual of matrix *A*, matrix $A^{d}$:

$\left(\begin{array}{ccc} \frac{1}{3} & \frac{1}{3} & \frac{1}{3}\\ 0 & 0 & 1\\ \frac{1}{2} & \frac{1}{4} & \frac{1}{4} \end{array}\right)$.

Matrixes *A* and $A^{d}$ define ergodic Markov chains. Both $\lim_{n\to \infty }A^{n}$ and $\lim_{n\to \infty }{\left(A^{d}\right)}^{n}$ are equal to: $\left(\begin{array}{ccc} 1/3 & 2/9 & 4/9\\ 1/3 & 2/9 & 4/9\\ 1/3 & 2/9 & 4/9 \end{array}\right)$.

Each row is equal to the equilibrium distribution π={1/3,2/9,4/9}=y. For both channels, we have y=Ay and $y=A^{d}y$.

### Appendix A.2. Information Channel

The matrix T/t can be considered a joint distribution p(X,Y) of random variables *X* input, and *Y* output, equally distributed,p(X)=p(Y)=y. The joint distribution p(X,Y) is the normalized matrix *T* by the total *t*: $\left(\begin{array}{ccc} \frac{1}{9} & \frac{1}{9} & \frac{1}{9}\\ 0 & 0 & \frac{2}{9}\\ \frac{2}{9} & \frac{1}{9} & \frac{1}{9} \end{array}\right)$ Matrixes *A* and $A^{d}$, together probability distribution of input p(X)=y and output p(Y)=y, define an information channel and its dual. The conditional probabilities are given by p(Y|X)=A and $p\left(X|Y\right)=A^{d}$.

The entropy of input and output is H(X)=H(Y)=H(y)=H({1/3,2/9,4/9})=1.53049.

H(X,Y) is the entropy of joint distribution p(X,Y) distribution, $H\left(X,Y\right)=-\sum_{i}\sum_{j}p_{ij}\log p_{ij}=\;2.72548$.

We can compute now I(X;Y) based on the equality H(X,Y)=H(X)+H(Y)−I(X,Y), and on the fact that in our channel H(X)=H(Y). We have I(X;Y)=2H(X)−H(X,Y)=0.3355.

The entropy of both channels is equal to H(Y|X)=H(X|Y)=H(X)−I(X,Y)=1.19499.

We compute now the entropies of the rows of *A* and $A^{d}$. For the first row of *A*: $H\left(Y|1\right)=H(\left\{\frac{1}{3},0,\frac{2}{3}\right\})=0.918296$, second row, $H\left(Y|2\right)=H(\left\{\frac{1}{2},0,\frac{1}{2}\right\})=1$, third row $H\left(Y|3\right)=H(\left\{\frac{1}{4},\frac{1}{2},\frac{1}{4}\right\})=1.5$. Observe that the entropy of the channel H(Y|X) can be obtained as the weighted average of the entropies of the rows: $H\left(Y|X\right)=\sum_{i}y_{i}H\left(Y|i\right)=1/3\times 0.918296+2/9\times 1+4/9\times 1.5=1.19499$.

For the first row of $A^{d}$: $H\left(X|1\right)=H(\left\{\frac{1}{3},\frac{1}{3},\frac{1}{3}\right\})=1.58496$, second row, H(X|2)=H({0,0,1)})=0, third row $H\left(X|3\right)=H(\left\{\frac{1}{2},\frac{1}{4},\frac{1}{4}\right\})=1.5$.

The entropy of the channel H(X|Y) can be computed too as $H\left(X|Y\right)=\sum_{i}y_{i}H\left(X|i\right)=1/3\times 1.58496+2/9\times 0+4/9\times 1.5=1.19499$. We see that H(Y|X)=H(X|Y).

Let us compute now the mutual information of the rows for *A* and $A^{d}$ channels. Remember that the mutual information of a row is the KL distance of this row to the output distribution, in our case y. For the first row of *A*: $I\left(1;Y\right)=D_{KL}\left(\left\{\frac{1}{3},0,\frac{2}{3}\right\},\left\{\frac{1}{3},\frac{2}{9},\frac{4}{9}\right\}\right)=0.38997$, second row, $I\left(2;Y\right)=D_{KL}\left(\left\{\frac{1}{2},0,\frac{1}{2}\right\},\left\{\frac{1}{3},\frac{2}{9},\frac{4}{9}\right\}\right)=0.37744$, third row $I\left(3;Y\right)=D_{KL}(\left\{\frac{1}{4},\frac{1}{2},\frac{1}{4}\right\},\left\{\left\{\frac{1}{3},\frac{2}{9},\frac{4}{9}\right\}\right)=0.27368$. Observe that mutual information I(X;Y) can be obtained as a weighted average of the mutual information of the rows: $I\left(X;Y\right)=\sum_{i}y_{i}I\left(i|Y\right)=0.38997\times 1/3+0.37744\times 2/9+0.27368\times 4/9=0.3355$.

For the $A^{d}$ channel, first row: $I\left(X;1\right)=D_{KL}\left(\left\{\frac{1}{3},\frac{1}{3},\frac{2}{3}\right\},\left\{\frac{1}{3},\frac{2}{9},\frac{4}{9}\right\}\right)=0.05664$, second row, $I\left(X;2\right)=D_{KL}\left(\left\{0,0,1\right\},\left\{\frac{1}{3},\frac{2}{9},\frac{4}{9}\right\}\right)=1.16993$, third row $I\left(X;3\right)=D_{KL}\left(\left\{\frac{1}{2},\frac{1}{4},\frac{1}{4}\right\},\left\{\frac{1}{3},\frac{2}{9},\frac{4}{9}\right\}\right)=0.12744$. Observe that mutual information I(X;Y) can be obtained as a weighted average of the mutual information of the rows: $I\left(X;Y\right)=\sum_{i}y_{i}I\left(X|i\right)=0.05664\times 1/3+1.16993\times 2/9+0.27368\times 4/9=0.3355$.

In Table A1 we show the values of entropy of source, entropy of channel, joint entropy and mutual information, which are equal for both channel *A* and its dual $A^{d}$, in Table A2 the values of entropies and mutual informations for both *A* and $A^{d}$ channels, plotted in Figure A1.

**Table A1:** The values of entropy of source, entropy of channel, joint entropy and mutual information for both channel *A* and its dual $A^{d}$.

Entropy of source H(X)=H(Y)	1.53049
Entropy of channel H(Y|X)=H(X|Y)	1.19499
Joint entropy H(X,Y)	2.72548
Mutual information I(X;Y)	0.3355

**Table A2:** The values of entropies and mutual informations of both channels *A* and its dual $A^{d}$ for each row in our toy example.

Row	1	2	3
Entropy of row	0.918296	1	1.5
Mutual information of row	0.38997	0.37744	0.27368
Entropy of row (dual)	1.58496	0	1.5
Mutual information of row (dual)	0.05664	1.16993	0.12744
